# Monthly Periscope

**Published:** 1853-07

**Authors:** 


*Monthly Periscope.*—We propose to furnish our readers monthly with a brief summary of current medical events; gathering together, and bringing to their notice the various facts, theories, and controversies—the floating waifs—which we find on the sea of medical journalism; giving to each that importance and prominence which it occupies in our own mind, and commenting upon them in such an informal way as our own views may indicate.

Dr. Lawson, in the Western Lancet for May, translates a paper from the French of M. Leudet, on the subject of Portal Phlebitis. Our readers will recollect the death, by this disease, of Dr. John Kearny Rodgers, of New York, a few months since. Since that time the attention of the profession has been called to this disease, first by some severe criticisms upon the diagnosis and treatment of that particular case; and later, by frequent mention, and reports of cases in the medical journals. In all these discussions the question has arisen as to our ability to diagnosticate the disease during life. It would seem that portal phlebitis has no symptoms peculiar to itself, and that as yet the limited number of cases does not permit of any grouping of symptoms for diagnostic purposes. Dr. Leudet's case, with its accompanying analysis of other reported cases, does not add to our means of discrimination. By his showing, no one symptom occurs in all cases, and though we may make a happy guess at the difficulty, we cannot rest on our diagnosis with that assurance which we feel in lung diseases. Tenderness in the region affected varies very much in its degree, and is not confined to the peculiar habitat of the disease. Chill, which has thus far been constant, is so common in other diseases, and is in this so irregular in the time of its manifestation, as to lose much of its diagnostic value. Again, we have no doubt that cases of portal phlebitis *may* occur without this symptom. Chill has usually been regarded as the unfailing precursor of abscess, but very recently we have witnessed the formation of a large pleuritic abscesss without chill, and without any manifestation by which the time of effusion could be ascertained or even guessed at. Icterus also occurs in only a portion of the cases. In a word, the phenomena of portal phlebitis are also those of peritonitis, whether idiopathic or occurring in the course of fevers. It is a sorry consolation to know that thus far portal phlebitis having been almost, if not quite uniformly fatal, the same importance does not attach to its diagnosis, as in a disease more within the reach of remedies. So many are the difficulties of its diagnosis and treatment, that opinions about it should be carefully expressed, and held *sub judice.* Neither would we criticise too closely that observer, who should mistake its nature, and err as to its treatment.

In all the Journals, and in all medical mouths, we hear of manifold new applications of quinine in therapeutics. This drug is assuming that paramount importance conceded some years ago to calomel, while the latter, shorn of its whilom honors, hides its diminished head. It would be shocking to the sensibilities of those of the olden school who now sleep with their fathers, could they behold the daring innovations now making in the treatment of phlogistic disease. Whether we shall come to give quinine in ten grain doses in pneumonia is, perhaps, doubtful, but would not be very surprising. Just now the use of this drug, in acute rheumatism, is on the tapis. At the recent meeting of the Monroe County Medical Society, Prof. E. M. Moore made some inquiries as to the knowledge of members of its exhibition in that disease. Two or three were found to have made trial of it, and that with very satisfactory results. In our own practice, we have a vivid recollection of the gratitude of an old fellow who had kept his bed for many weeks with a subacute rheumatism of most distressing severity. He took quinine twice daily, in six grain doses, with a great mitigation of his sufferings, and a corresponding improvement of the general health. Coming home from town meeting, however, with an unusually large brick in his hat, my friend, singularly enough for a man in his condition, became hydropathic, and composed himself to sleep in a run of water by the roadside. This was on the first Tuesday in March. A renewed attack followed this slight imprudence, and he again had cause to bless the Society of Jesus for the introduction of quinine into our therapeutics. But in cases of sciatic rheumatism have we seen the happiest effects, and on our recent removal from our old field of labor, several patients came to us for a copy of the prescription from which they had derived such striking benefit.

The Charleston Medical and Surgical Journal has a monograph by Dr. La Roche, on malaria. He demonstrates the existence of such a cause of disease by a host of facts, involving much research and labor. It is an article deserving praise for the care given to its preparation. The wonder in the matter is, that it should be necessary to revive this discussion of the effects of paludal exhalations. Very recently, however, Dr. Dundas, (of quinine in typhus celebrity,) has denied the existence of marsh malaria as a cause of disease. It seems to us that only one part of this question is open. *Is vegetable decay a necessary condition for the production of marsh miasm?* Dr. Ferguson's arguments, based upon the ill-fated Walcheren expedition, and the military operations in the peninsular war, have never been contradicted. For on dry sandy plains, destitute of vegetation, dry on the surface, but lying low, and having water a little distance down, we find those fevers usually considered miasmatic, prevailing with dysentery to a frightful extent. The sandy plain of Alentejo, on the opposite side of the Tagus from Lisbon, is a familiar instance of this. A night passed there is sure to be followed by fever, while across the river, two miles in width, is a rich garden country of luxuriant vegetation, but still very healthy. Vegetable decomposition exists everywhere, and were it alone the cause of malaria, would produce it in every place where vegetation exists. These views are somewhat different from those previously entertained by us — but the point of difference does not rest upon the question of malaria or non-malaria, neither can we yet explain all those American facts, incident to the flooding of swamps, and the clearing of forests. This is an enticing subject, and its importance will justify frequent reference to it.

*Diabetes.*— Dr. Wood, in the Transactions of the Philadelphia College of Physicians, relates the history of a case of diabetes, in which yeast was exhibited with a gratifying measure of success. It was given as a ferment, with the idea of decomposing the sugar in the blood. This is not entirely new. Dr. Watson relates a case or two of its administration, but he was obliged to suspend it on account of the fermentation produced in the stomach causing great distention. His patients complained of being blown up like a bladder of wind. On second thought, however, we believe that the yeast was given as a chemical test of the existence of sugar in the stomach, he having previously withheld saccharine food from his patients, and not with a view to its therapeutic effect. Dr. Wood's case runs only through two weeks, an insufficient time for any reliable test. Dr. W. alludes to the use of rennet by Dr. Gray, of Glasgow, Scotland, which our readers may have noticed elsewhere. A table-spoonful of rennet was given three times daily. Rennet out of the body, has the property of converting sugar into lactic acid, and was given with the hope of producing the same effect in the system. At the end of six months, there was a mere trace of sugar in the urine, and the general health was much improved. This is one of the rare instances of *a priori* reasoning correctly applied to therapeutics. When we come to understand thoroughly the chemistry of the fluids, we shall have many such.

We have one or two questions to ask of the cavilers against the originality of Dr. Reid's method of reducing luxations of the hip-joint:

1st. Did you yourself, personally, previously to Dr. Reid's publication, ever hear, or read, or even dream, of such a method?

2d. If some "distinguished metropolitan surgeon," "one of our set," living either in Paris, London, or New York, had announced the method, would it not have been a purely original, and most ingenious discovery? And should we have heard one word of the "lamented Dr. Nathan Smith," whose *caveat* seems to cover every conceivable method of pulling at a dislocation; or, as a friend says, "all of God's creation, and part of Massachusetts?"

The St. Louis Medical and Surgical Journal has an article from Dr. Mattingly, on erysipelas, containing some noteworthy ideas. Dr. M. considers erysipelas an eruptive fever in the same sense as variola, varicella, or scarlatina. He deprecates any attempt to check the progress of the cutaneous inflammation, and looks upon a free eruption as the great safeguard of the internal organs. Of course this rules out in a great measure all local treatment, and simplifies the internal medication. Dr. M. is not alone in his views. In the last Braithwaite, Dr. Bennett, of Edinburgh, has a very able article on this same matter, and advocates a similar treatment — a sudorific, and cordial stimulant course. There is a query as to whether simple uncomplicated erysipelas requires medication to any greater extent than does rubeola. In the Hotel Dieu, cases of scalp erysipelas are left to nature, M. Louis asserting that they are never fatal. We propose at our leisure to enter into an analysis of this subject, and to base some conclusions on the various pathological views now entertained. Do any of our readers know aught of Dr.

Hamilton Bell's treatment by the tinct. ferri. muriatis. In a single case in our hands, where this was the only medication, the patient did well enough and we thought tha tthe eruption, which was somewhat extensive, was of a livelier red than usual. During the whole time of exhibition, the patient was in a profuse perspiration — an unexpected occurrence.

*Veratrum viride.*—Dr. Norwood's remedy will have notice. There is no keeping it down. Every one decries its virtues, while nobody knows anything about it, but as often as it is annihilated by the attacks of medical writers, so often does it rise again, a very Phœnix. It has been completely destroyed two or three times—dead and buried—but *Resurgam* is written on its tombstone. Will not some one treat a few pneumonias and typhoid fevers with it, and inform us of the result? No common pneumonia will die under it, unless the remedy is positively hurtful, and we should like to see this phantom laid. "It will not down," so we must ascertain its true value, and find a place for it in our already too cumbersome armamentaria. A Chautauque county epistolary friend promised us some information. Did it kill anybody, Dr. S.?

Our western editorial brethren are given to controversy. Dr. S. Hanbury Smith has said some good natured things about Hahnemann, giving him credit for some indirect, and unintentional benefit to the world and the profession, by unwittingly teaching us that the *vis medicatrix naturæ* is something more than an *umbra nominis.* Dr. Lawson, of the Western Lancet, deprecates any such soft handling. He goes in for war to the knife, and does not concede to Hahnemann the rights of honorable warfare. But if Dr. Smith has sinned grievously, in what category shall we place the Massachusetts Medical Society; which holds in its maternal bosom sundry practitioners of the infinitesmal enormity? Nice men they are too, having had the great advantages of the University, A. B.'s, and M. D.'s in regular standing. There seems to be the difficulty. They are very gentlemanly quacks, and not to be classed with those who sin through ignorance. Give our somewhile competitor, Dr. Walters, the celebrated "witch doctor," of Canandaigua, an education, dress him in broadcloth, polish up his boorishness and reform his profanity, but leave him the same unmitigated scamp he now is, and according to this scale of qualification, he would not merit expulsion from the oldest, most venerable, and most thoroughly organized of our state societies. S. B. H.

